# Clinical utility of polygenic risk scores: a critical 2023 appraisal

**DOI:** 10.1007/s12687-023-00645-z

**Published:** 2023-05-03

**Authors:** Sebastian Koch, Jörg Schmidtke, Michael Krawczak, Amke Caliebe

**Affiliations:** 1 Institut für Medizinische Informatik und Statistik, Christian-Albrechts-Universität zu Kiel, Universitätsklinikum Schleswig-Holstein Campus Kiel, Kiel, Germany; 2 Amedes MVZ Wagnerstibbe, Hannover, Germany; 3 Institut für Humangenetik, Medizinische Hochschule Hannover, Hannover, Germany

**Keywords:** Genetic risk, Heritability, Diagnostics, Prognostics, Validation, Clinical score

## Abstract

Since their first appearance in the context of schizophrenia and bipolar disorder in 2009, polygenic risk scores (PRSs) have been described for a large number of common complex diseases. However, the clinical utility of PRSs in disease risk assessment or therapeutic decision making is likely limited because PRSs usually only account for the heritable component of a trait and ignore the etiological role of environment and lifestyle. We surveyed the current state of PRSs for various diseases, including breast cancer, diabetes, prostate cancer, coronary artery disease, and Parkinson disease, with an extra focus upon the potential improvement of clinical scores by their combination with PRSs. We observed that the diagnostic and prognostic performance of PRSs alone is consistently low, as expected. Moreover, combining a PRS with a clinical score at best led to moderate improvement of the power of either risk marker. Despite the large number of PRSs reported in the scientific literature, prospective studies of their clinical utility, particularly of the PRS-associated improvement of standard screening or therapeutic procedures, are still rare. In conclusion, the benefit to individual patients or the health care system in general of PRS-based extensions of existing diagnostic or treatment regimens is still difficult to judge.

**Supplementary Information:**

The online version contains supplementary material available at 10.1007/s12687-023-00645-z.

## Introduction

Genome-wide association studies (GWAS), as performed in large numbers over the last 20 years, have proven the genetic architecture of most, if not all, common human diseases to be complex. Contrary to original expectation (Reich & Lander 2001), the heritability of diseases such as cancer or diabetes was not found to be explicable by a handful of common genetic variants with strong effects (Lewis & Vassos 2020; Slunecka et al. 2021). Instead, GWAS of common single nucleotide polymorphisms (SNPs) yielded hundreds to thousands of weak to moderate disease associations, and even an “omnigenic model” has been proposed according to which variation in all genes expressed in disease-relevant human cells plays a potential role in common complex disease etiology (Boyle et al. 2017).

One way to aggregate the joint effects of a large number of SNPs upon the risk of a common complex disease is by way of so-called ‘polygenic risk scores’ (PRSs). PRSs sum up a large number of single-variant association statistics so as to combine these (individually weak) effects in a single number for use in disease diagnosis, prognosis or treatment, and in research (Lambert et al. 2019). In the process, the computation of a PRS mostly draws upon summary statistics of large GWAS, which may be shared without raising data privacy concerns (Thelwall et al. 2020).

There are several different methods to construct a PRS. The most straightforward approach is to include all known, clinically relevant risk variants for a given disease, such as the *BRCA* gene mutations for breast cancer, supplemented perhaps by genome-wide significant hits from GWAS. In this case, however, the number of SNPs would usually be small, implying that the resulting PRS cannot capture much of the polygenic nature of the disease in question. More recent methods of PRS construction also include SNPs with disease associations lacking genome-wide significance. However, owing to their sheer number, many of these SNPs will be in linkage disequilibrium (LD) with each other so that simple summation of their association statistics in a PRS would be inappropriate. The most common methods to deal with this problem are clumping-and-thresholding, Bayesian inference and penalized regression (Choi & O’Reilly 2019; Ge et al. 2019; Lloyd-Jones et al. 2019; Mak et al. 2017; Prive et al. 2020). While the first removes SNP-SNP correlations by keeping only the most significant SNPs representative of an LD cluster, the other two approaches usually downweigh the effect sizes of individual SNPs before their inclusion into a PRS, taking the local strength of LD into account.

PRSs are calculated at the level of the individual and therefore are ascribed potential utility for ‘precision’ medicine, especially in terms of disease prognosis at an early age, if not at birth or even before (Choi et al. 2020; Slunecka et al. 2021). However, the strong role of environmental and lifestyle factors in the etiology of common complex diseases, which inherently cannot be captured by a PRS, has inevitably dimmed the hope for a substantial contribution of PRSs to precision medicine (Caliebe et al. 2021; Herzig et al. 2022). Since heritability equals the proportion of population-level phenotypic variability explicable by genetic variability (Genin 2020; Visscher et al. 2008), the diagnostic and prognostic performance of a PRS is conceptually limited by the heritability of the phenotype in question when there is no gene-environment correlation. Worthy of note in this context, heritability is not the same as genetic causality. The latter may, in fact, be overestimated by the heritability of a phenotype when there is gene-environment correlation due to, for example, population stratification or the shared environment of blood relatives (Young et al. 2018). In such instances, it is conceivable that the diagnostic or prognostic performance of a PRS exceeds that from direct genetic causality. Moreover, heritability is not a natural constant but depends upon the population specifics of disease-relevant genetic and environmental factors. Finally, heritability says nothing about the genetic architecture of a given phenotype, neither in terms of the number nor the effect sizes of phenotype-relevant genetic variants, nor of the interaction of the latter with each other or with the environment.

Generally, the heritability of common complex diseases in humans is much smaller than 100% (Schork 1997) so that, by definition, a PRS cannot fully explain the presence or absence of such a disease as long as environmental and lifestyle factors are disregarded. What is more, classic PRSs only comprise common genetic variants (e.g. SNPs) that were found to be disease-associated in GWAS. This implies that the contributions of rare variants and gene–gene-interactions to disease risk are not adequately represented as well. In consequence, it may be concluded that the diagnostic and prognostic performance of a PRS will not even come close to the heritability of the target disease.

The present study aims at providing an up-to-date overview of the current prospects of PRSs for clinical practice. Instead of representing a fully comprehensive review of the literature on PRSs, however, we exemplify PRS-disease relationships and their potential for diagnosis, prognosis and therapeutic decision making for selected diseases, representing a broad spectrum of clinical conditions (cancer, neurological, psychiatric, internal).

## Materials and methods

### Study retrieval and information about PRSs

We focused upon PRSs constructed for one of nine common complex diseases: breast cancer, type 1 diabetes, type 2 diabetes, prostate cancer, coronary artery disease, Parkinson disease, Alzheimer disease, major depressive disorder and schizophrenia. Heritability estimates for these diseases were derived from European twin studies, excluding studies that were based exclusively upon Finns. The studies were retrieved from PubMed or Google using search term ‘heritability’ AND [disease].

We first investigated PRSs constructed from case–control studies to assess their specific *diagnostic* performance (Table 1). We confined our study to PRSs that were based upon European ancestry GWAS, again excluding Finns. In addition to drawing upon the polygenic score catalogue (Lambert et al. 2020) and selected review articles (Byrne & Toland 2021; Chatterjee et al. 2016; Fullerton & Nurnberger 2019; Lambert et al. 2019; Lewis & Vassos 2020; Padilla-Martinez et al. 2020; Slunecka et al. 2021; Yanes et al. 2020; Zeinomar & Chung 2021), we searched PubMed and Google with terms (‘PRS’ OR ‘polygenic risk score’ OR ‘polygenic score’) AND [disease] as well as ‘validation’ AND (‘AUC’ OR ‘AUROC’). The results were narrowed down to publications that provided sufficient details about the respective PRS, including the number of SNPs and the method of PRS construction. In addition, the diagnostic performance of a PRS must have been quantified by the area under curve (AUC) for the PRS alone (i.e. without adjustment for additional variables). Owing to these limitations, our study cannot claim completeness or being fully systematic. Moreover, we focused upon PRSs that were as general as possible for the disease in question in the sense that they were not specific, for example, for a disease subtype or a certain age at onset.

**Table 1:** Diagnostic performance of PRSs for exemplary common complex diseases

Disease	Heritability	Largest GWAS to date (# cases/ # controls)	SNP selection method	Sample size (# cases/ # controls)		# SNPs (PRS)	AUC original	AUC validation	Reference
				Discovery dataset	PRS generation dataset				
Alzheimer disease	58%^1^	39,106 + 46,828 (proxy) /401,577(Bellenguez et al. 2022)	risk + GWS	n.a (2 APOE SNPs)	n.a	2	0.688	-	Escott-Price et al. (2015)
			risk + GWS	25,580/ 48,466^2^	n.a	22	0.715	-	
			clumping + thresholding	13,831/ 29,877^2^	3,177/ 7,277	87,605	0.745	-	
			clumping + thresholding	17,008/ 37,154^2^	1,011/ 583	205,068	0.84^3^	-	Escott-Price et al. (2017)
			penalized regression	538/269,166		15	0.723	-	Tanigawa et al. (2022)
Breast cancer	31%^4^	122,977/ 105,974 (Michailidou et al. 2017)	risk + GWS	9,895/ 259,809^5^	33,673/ 33,381	77	-	0.612^6^	Mavaddat et al. (2015)
			risk + GWS, clumping + thresholding	88,916/69,732		313	0.639	0.628^7^	Mavaddat et al. (2019)
			penalized regression	88,916/ 69,732	5,159/ 5,285	3820	0.646	-	
			Bayesian	122,977/ 105,974^8^	2,576/ 60,771	5218	-	0.613^6^	Khera et al. (2018)
			risk + GWS	15 studies	n.a	76	0.68	-	Vachon et al. (2015)
			penalized regression	9,895/259,809		555	0.596	-	Tanigawa et al. (2022)
Coronary artery disease	57% (male)^9^ 38% (female)^9^	190,493/582,775 (Tcheandjieu et al. 2022)	metaPRS	metaPRS from 3 studies		1,745,180	-	0.636^10^ 0.502 (DE)^11^ 0.660 (EB)^11^ 0.638 (UKB)^11^	Inouye et al. (2018)
			Bayesian	60,801/ 123,504^12^	3,963/ 116,317	6,630,150	-	0.634^10^ 0.670 (DE)^11^ 0.562 (EB)^11^ 0.637 (UKB)^11^	Khera et al. (2018)
			clumping + thresholding	60,801/ 123,504^12^	7,912/ 121,941	300,238	0.640	-	Bolli et al. (2021)
			metaPRS	metaPRS from 2 studies		1,926,521	0.645	-	
			metaPRS	metaPRS from 2 studies		6,695,156	0.641	-	
			penalized regression	60,801/ 123,504^12^	15,947/ 15,947	40,079	0.629	^−^	Elliott et al. (2020)
			Bayesian	60,801/ 123,504^12^	4,746/ 88,182	2,994,055	0.643	-	Ye et al. (2021)
			clumping + thresholding	60,801/ 123,504^12^	471/ 9,529	1940	0.613	0.605 (DE)^11^ 0.514 (EB)^11^	Gola et al. (2020)
				60,801/ 123,504^12^	308/ 9,692	375,822	0.657	0.541 (DE)^11^ 0.604 (UKB)^11^	
				60,801/ 123,504^12^	5,302/ 4,698	3,423,987	0.675	0.616 (EB)^11^ 0.599 (UKB)^11^	
Major depressive disorder	37%^13^	170,756/329,443 (Yao et al. 2021)	Bayesian	248,750/ 563,184^14^	max 3,760 (cross validation)	-	0.601	-	Ni et al. (2021)
			clumping + thresholding	16,301/ 50,870^15^	14,696/ 22,013	-	0.56	-	Cai et al. (2020)
Parkinson disease	27%^16^	56,306/ 1,417,791 (Nalls et al. 2019)	clumping + thresholding	56,306/ 1,417,791^17^	5,851/ 5,866	1805	0.64	0.692^17^ 0.645^18^	Nalls et al. (2019)
			risk + GWS	20,184/ 397,324^19^	n.a	43	0.616^20^	-	Bobbili et al. (2020)
Prostate cancer	58%^21^	79,148/61,106 (Schumacher et al. 2018)	risk + GWS	13 studies > 500/500	n.a	72	0.64	-	Black et al. (2020)
			risk + GWS, clumping + thresholding	risk + GWS: 21 studies clumping + thresholding: 12,153/13,003		133	0.68	-	Szulkin et al. (2015)
			risk + GWS	79,148/ 61,106^22^	n.a	147		0.662^23^	Schumacher et al. (2018)
			penalized regression	6,278/263,426		948	0.640	-	Tanigawa et al. (2022)
Schizophrenia	79%^23^	40,675/64,643 (Pardinas et al. 2018)	Bayesian	31,328/ 41,191^24^	max 7,763 (cross validation)	-	0.734	-	Ni et al. (2021)
			clumping + thresholding	> 33,356/ > 43,724^25^	53/ 9,151	79,837	0.71	-	Zheutlin et al. (2019)
			Bayesian	> 33,356/ > 43,724^25^	53/ 9,151	971,463	0.74	-	
Type 1 diabetes	72%^26^	18,942/501,638 (Chiou et al. 2021)	risk + GWS, Bayesian	2 studies	4,574/ 1,207	41	0.87	0.84^27^	Winkler et al. (2014)
			risk + GWS	5 studies	n.a	30	-	0.886^28^ 0.893^28^	Oram et al. (2016)
			risk + GWS	10 studies	478/ 290	37	0.86	-	Perry et al. (2018)
			risk + GWS	> 5 studies	6,483/ 9,246	67	0.927	0.921^29^	Sharp et al. (2019)
			penalized regression	286/269,418		69	0.765	-	Tanigawa et al. (2022)
Type 2 diabetes	26%^30^, 72%^31^	148,726/965,732 (Vujkovic et al. 2020)	clumping + thresholding	12,171/ 56,862^32^	182,422	25,454	0.795	-	Liu et al. (2021)
			Bayesian	26,676/ 132,532^33^	4,639/ 88,289	2,996,761	0.645	-	Ye et al. (2021)
			penalized regression	2,188/267,516		385	0.576	-	Tanigawa et al. (2022)

Largest GWAS to date: GWAS with the largest number of cases. For consistency with the PRS selection criteria used in our study, the above survey was limited to GWAS of populations of European ancestry. # cases/ # controls: number of cases/number of controls, discovery dataset: dataset used for SNP selection and/or a priori variant effect size estimation (usually from the summary statistics of GWAS), PRS: polygenic risk score, SNP: single nucleotide polymorphism, # SNP (PRS): number of SNPs included in the PRS, GWS: genome-wide significant, AUC: area under the receiver operating curve, AUC original: AUC obtained in the original study (column ‘reference’), AUC validation: AUC obtained in an independent dataset, DE: German dataset, EB: Estonian biobank, UKB: UK Biobank,

^1^Gatz et al. (2006), ^2^Lambert et al. (2013), ^3^data overlap with GWAS, but AUC corrected for overlap, ^4^Mucci et al. (2016), ^5^Michailidou et al. (2013), ^6^Mavaddat et al. (2019), ^7^Jia et al. (2020), ^8^Michailidou et al. (2017), ^9^Zdravkovic et al. (2002), ^10^Bolli et al. (2021), ^11^Gola et al. (2020), ^12^(Nikpay et al. 2015), ^13^Sullivan et al. (2000), ^14^(Wray et al. 2018) and UKB, ^15^ (Cai et al. 2020), ^16^Goldman et al. (2019), ^17^Nalls et al. (2019), ^18^Koch et al. (2021), ^19^Chang et al. (2017), ^20^sex as covariate in AUC computation, ^21^Hjelmborg et al. (2014), ^22^Schumacher et al. (2018), ^23^Jia et al. (2020), ^23^Hilker et al. (2018), ^24^Pardinas et al. (2018), ^25^Schizophrenia Working Group of the Psychiatric Genomics (2014), ^26^Kyvik et al. (1995), ^27^Winkler et al. (2014), ^28^Sharp et al. (2019), ^29^(Sharp et al. 2019), ^30^Poulsen et al. (1999), ^31^Willemsen et al. (2015), ^32^Morris et al. (2012), ^33^Scott et al. (2017).

We classified the validation of a given PRS in an independent dataset as ‘external’ when it was performed by authors other than those of the original report, or as ‘internal’ otherwise. The PRS construction methods were classified into five categories. Methods using only known clinically relevant risk SNPs and genome-wide significant SNPs were labeled ‘risk + GWS’. Approaches that first pruned or clumped SNPs by LD, and then selected SNPs with a p value of the GWAS summary statistic below a certain threshold, were labelled ‘clumping + thresholding’. When several existing PRSs were combined into a single PRS, this PRS was labelled ‘metaPRS’. If the PRS weights had been adjusted with Bayesian methods, the approach was termed ‘Bayesian’. Finally, the adjustment of weights by penalized regression, such as Lasso, was labelled ‘penalized regression’.

Next, we searched for PRSs constructed in a *prognostic* context from, or applied to, prospective cohort studies. The search procedure was very similar to that followed above for the diagnostic setting. As a sole exception, term (‘AUC’ OR ‘AUROC’) was replaced by (‘Harrell ‘s C’ OR ‘C statistic’). The C statistic measures the goodness-of-fit of risk score-based statistical models of right-censored survival time data. Similar to the AUC, the value of the C statistics can range from 0.5 to 1, with a value of 1 indicating that the risk score perfectly predicts which of two individuals develops the target disease first. We only included studies that reported C statistics.

### Combination of PRSs with clinical risk scores

We examined the effect of combining a PRS with a clinical risk score upon the predictive performance of both. This analysis was first performed for breast cancer because exceptionally many clinical risk scores have been developed for this disease. Studies were retrieved from reviews by Fung et al. (2019) and Lambert et al. (2019) as well as through PubMed and Google searches for (‘PRS’ OR ‘polygenic risk score’ OR ‘polygenic score’) AND [breast cancer score]. We confined our analysis to PRSs that comprised at least 15 SNPs.

Selected combinations of a PRS and a clinical risk score were then also considered for less well covered conditions, including prostate cancer, Parkinson disease, coronary artery disease and type 2 diabetes. The respective studies were either retrieved from reviews by Lambert et al. (2019) and Byrne & Toland (2021), or through PubMed and Google searches for (‘PRS’ OR ‘polygenic risk score’ OR ‘polygenic score’) AND (‘model’ OR ‘complex’) AND [disease]. Other than in our analysis of PRSs alone, AUC and C statistic were allowed to have been adjusted for clinical covariates because we were only interested in comparing the predictive performance of the PRS with and without the clinical risk score in the same cohort.

### Statistical analysis

Linear regression analysis was performed for the PRSs listed in Table 1 treating the estimated heritability of the disease in question as the explanatory variable and the AUC of the PRS as the response variable. For coronary artery disease, we considered the average of the two gender-specific heritability estimates reported in the literature. The regression analysis was performed using the *lm* command of R version 4.1.3 (R Core Team 2022). The results were visualized with the *geom_smooth* function of *ggplot2* version 3.3.5 (Wickham 2016), setting the *method* parameter to ‘lm’.

All plots were generated with *ggplot2* version 3.3.5.

## Results

### Heritability

Of the exemplary common complex diseases for which PRSs have been retrieved from the literature (Table 1), Parkinson disease, breast cancer and major depressive disorder had the lowest heritability (27%, 31% and 37%, respectively). Heritability estimates for coronary artery disease (sex average: 48%), Alzheimer disease and prostate cancer (both 58%) were found to be intermediate whilst the highest values have been reported for type 1 diabetes (72%) and schizophrenia (79%). Notably, the heritability estimates for type 2 diabetes varied considerably between studies (26% to 72%) and, for coronary artery disease, the heritability was found to be higher for men (57%) than women (38%) (Zdravkovic et al. 2002).

### Diagnostic performance of PRSs

We first investigated how well PRSs could differentiate between cases and controls, i.e. how well they performed in a *diagnostic* context. The most frequently applied method of PRS construction was the use of known, clinically relevant risk alleles and genome-wide significant SNPs (‘risk + GWS’). In these cases, the ensuing PRS typically comprised < 100 SNPs. LD clumping combined with p value thresholding (‘clumping + thresholding’) was the second most frequent approach and typically included thousands to tens of thousands of SNPs into the PRS. Bayesian selection (‘Bayesian’) was the next most frequently used method and often resulted in the utilization of up to several million SNPs. Penalized regression (‘penalized regression’) was used in three studies whilst a metaPRS was developed in two studies (‘metaPRS’).

The diagnostic performance of PRSs was found to vary considerably (Table 1), with AUC values ranging from 0.502 for coronary artery disease (Inouye et al. 2018) to 0.927 for type 1 diabetes (Sharp et al. 2019). Note that, here and in the following, all performance measures are given with original precision or were rounded to three decimal places if the original precision was higher. Even for one and the same disease, the AUC was sometimes highly variable. For coronary artery disease, for example, the AUC of PRSs ranged for 0.502 to 0.675 while, for breast cancer, the lowest AUC was 0.596 and the highest AUC was 0.68. At least in part, this variation may be explicable by the use of different cohorts for internal and external validation. Thus, in a study by Gola et al. (2020) of two PRSs for coronary artery disease, the AUC of the PRS of Inouye et al. (2018) varied between 0.502 and 0.660, and that of the PRS of Khera et al. (2018) between 0.562 and 0.670, in the same European ancestry cohorts. Tanigawa et al. (2022) used a method specifically designed to obtain “sparse” PRSs from comparatively small numbers of SNPs, and developed 813 such PRSs from the UK Biobank data. Notably, the UK biobank is population-based and hence provides only few cases for many diseases. This limitation may explain why the PRSs reported by Tanigawa et al. (2022) performed poorer, on average, than other PRSs based upon larger numbers of cases. Most PRS published so far were not based upon the largest GWAS datasets available at the time. Consideration of these resources and of additional data gathered, for example, by private companies like 23andMe could potentially improve the performance of PRS beyond the level shown in Table 1.

Usually, AUCs were lower in external validation studies than in original publications. However, one of the exceptions that proved the rule was the breast cancer PRS of Mavaddat et al. (2019), comprising 313 SNPs, for which a similar AUC was achieved in the original study (0.639) and the external validation (0.628) (Jia et al. 2020). For the Parkinson disease PRS of Nalls et al. (2019), the validation AUC (0.645) as reported by Koch et al. (2021) even slightly surpassed the original value (0.640). Only a few PRSs have been validated independently, namely the breast cancer PRS of Mavaddat et al. (2019), by Jia et al. (2020), and that of Khera et al. (2018), by Mavaddat et al. (2019), the coronary artery disease PRSs of Inouye et al. (2018) and of Khera et al. (2018), both by Bolli et al. (2021) and Gola et al. (2020), and the Parkinson disease PRS of Nalls et al. (2019), by Koch et al. (2021).

**Fig. 1:**
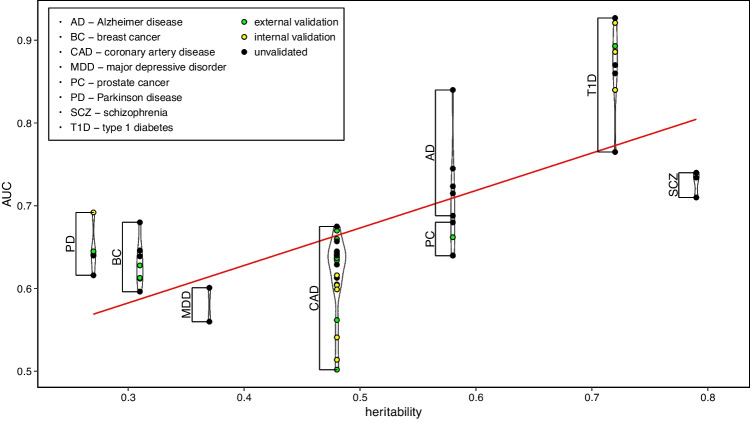
**Relationship between heritability and PRS diagnostic performance.** The violin plot relates disease-specific heritability estimates to the AUC of PRSs (see Table 1). A sex-averaged heritability estimate was considered for coronary artery disease; type 2 diabetes was excluded because of its widely varying heritability estimates. The red line was derived by linear regression analysis. PRS: polygenic risk score, AUC: area under the receiver operating curve

We next examined whether a higher heritability was associated with a better diagnostic performance of a PRS. While a trend towards higher AUC with increasing heritability indeed became apparent for the PRSs studied here, there was also considerable variability between diseases (Fig. 1). For example, the PRSs for Parkinson disease had remarkably high AUC despite a low heritability of the disease of only 27%. On the other hand, PRSs for major depressive disorder and schizophrenia yielded average AUC values that were notably smaller than for diseases of similar heritability. This variation likely reflects the above-mentioned fact that heritability does not account for the specific genetic architecture of a disease. If its heritability is mainly due to common variants, like those targeted by GWAS, a PRS may perform better than if the disease is mostly caused by rare variants or, possibly, gene–gene interactions. The same is true for diseases for which the number of causal variants is small, rather than heritability being distributed diffusely across the genome. Noteworthy, almost all common diseases have been shown to have a large polygenic component (O'Connor 2021), with only few exceptions, such as type I diabetes.

### Prognostic performance of PRS

In a prognostic context, PRSs would offer the specific advantage that, other than clinical or lifestyle parameters, they do not change over lifetime. However, the prognostic performance of a PRS will usually be worse than its diagnostic performance. This is because any meaningful PRS can be expected to be negatively correlated with the age at onset (AO) of the disease in question (Caliebe et al. 2021; Koch et al. 2021; Pavelka et al. 2022; Sleegers et al. 2015). Although potentially useful in its own right, this negative correlation implies that the relative lifetime risk of individuals with a low PRS is always higher than their relative frequency among cases, compared to controls. What is more, the stronger the negative correlation between PRS and AO, the larger the discrepancy between prognostic and diagnostic value of the PRS. Therefore, the prognostic performance of a PRS cannot be equated to its diagnostic performance in the underlying (case–control) GWAS, but must be determined in prospective studies before putting the PRS to practical prognostic use.

Despite these more general caveats, a small number of studies have already been published that developed or validated PRSs in past prospective cohorts, thereby validly, albeit retrospectively, addressing the prognostic performance of the PRSs (Table 2). Instead of the AUC, the C statistic for right-censored survival time data was included as performance measure in these reports (see Methods). Although the studies already gave a first indication of the prognostic performance of PRSs, more research is clearly necessary in this regard.

**Table 2:** Prognostic performance of PRSs for breast cancer and coronary artery disease

Disease	SNP selection	# SNPs (PRS)	C statistic	Reference
Breast cancer	risk + GWS	77	0.622	Mavaddat et al. (2015)
		88	0.541^1^ 0.566^2^	Kuchenbaecker et al. (2017)
Coronary artery disease	penalized regression	40,079	0.608	Elliott et al. (2020)

PRS: polygenic risk score, SNP: single nucleotide polymorphism, # SNP (PRS): number of SNPs included in the PRS, GWS: genome-wide significant, C statistic: Harrell’s C statistic, ^1^*BRCA1* mutation carriers, ^2^*BRCA2* mutation carriers.

### Combination of PRSs with clinical scores

Clinical (i.e. non-genetic) scores have been developed for many common complex diseases, and some of these scores are also being used for prognostics, particularly in the case of breast cancer. This includes the BOADICEA and the IBIS risk model, also known as the ‘Tyrer-Cuzick model’ (Lee et al. 2019; Tyrer et al. 2005). The clinical parameters normally included in breast cancer scores are family history, age, breast density, age at menarche and age at birth of the first living child. While some scores target both the risk of carrying a *BRCA* gene mutation and the risk of developing breast cancer, other scores are only applicable to *BRCA* non-carriers. Therefore, the consequences of a high score may vary from case to case, including testing for *BRCA* gene mutations or more frequent, or earlier, mammographic screening.

**Fig. 2:**
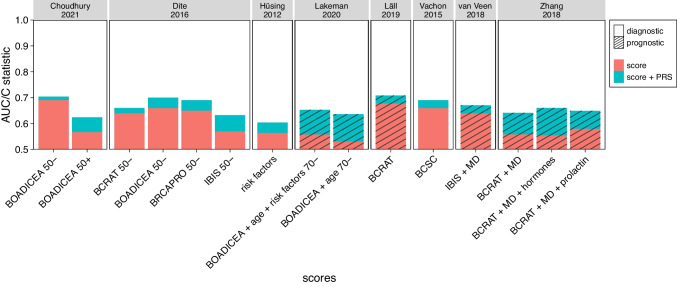
**Improvement of clinical scores for breast cancer by their combination with a PRS.** PRS: polygenic risk score, AUC: area under the receiver operating characteristic curve. For details on individual studies, see Supplementary Table S1. BOADICEA + risk factors (Choudhury et al. 2021) was excluded because no AUC was available. The y axis only starts at 0.5 because any smaller AUC would imply that the corresponding score is worse in assigning disease status (affected, not-affected) than flipping a coin

The improvement of the performance of a clinical score for breast cancer by its combination with a PRS would be reflected by an increase of either the AUC or the C statistic. In our survey (Supplementary Table S1, Fig. 2), we considered the BCRAT, BOADICEA, BRCAPRO, IBIS and BCSC scores (Gail et al. 1989; Parmigiani et al. 1998; Tice et al. 2008), in addition to the combination of various clinical parameters. The original studies took different approaches to combine clinical scores with PRSs, from simple multiplication (Dite et al. 2016; Vachon et al. 2015; van Veen et al. 2018), via the simultaneous inclusion of both scores in a logistic regression or Cox model (Husing et al. 2012; Lall et al. 2019; Zhang et al. 2018), to the direct inclusion of the early PRS of Mavaddat et al. (2015) into the BOADICEA score (Choudhury et al. 2021; Lakeman et al. 2020).

In general, combination with a PRS yielded only minor to moderate improvement of the respective clinical score. In some cases, the PRS alone performed nearly as well as the combination of clinical score and PRS (Supplementary Table S1). The largest improvement of the *prognostic* performance of a clinical score was reported for the combination of BOADICEA + age with the late PRS of Mavaddat et al. (2019), raising the C statistic from 0.531 to 0.636 (Lakeman et al. 2020). Note, however, that the C statistic of the PRS alone already ranged from 0.632 (age < 60 years) to 0.673 (age 60 to 70 years). The same PRS also yielded the smallest increase in *diagnostic* performance of all combinations considered. Thus, integrating the PRS into the BOADICEA formula raised the AUC of the latter from 0.691 to 0.704 for women under 50 years of age (Choudhury et al. 2021). The corresponding study by Choudhury et al. (2021) also revealed that BOADICEA had a better diagnostic performance for women < 50 years than for older women (AUC 0.691 vs. 0.568), and that combination with a PRS provided greater benefit to the latter group. The best *prognostic* performance of a combination between a clinical score for breast cancer and a PRS was obtained by adding the early PRS of Mavaddat et al. (2015) to the BCRAT model (Lall et al. 2019), raising the C statistics from 0.627 (PRS) and 0.677 (score) to a combined value of 0.708.

**Fig. 3:**
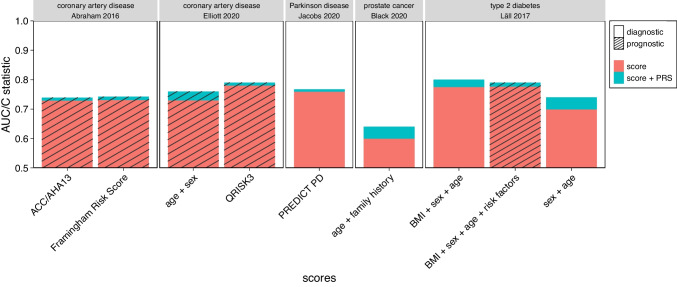
**Improvement of clinical scores, for selected diseases other than breast cancer, by their combination with a PRS**. PRS: polygenic risk score, AUC: area under the receiver operating characteristic curve. For details on individual studies, see Supplementary Table S2. The y axis only starts at 0.5 because any smaller AUC would imply that the corresponding score is worse in assigning disease status (affected, not-affected) than flipping a coin

We also addressed the effect of combining clinical scores or parameters with PRSs for prostate cancer, Parkinson disease, coronary artery disease and type 2 diabetes (Supplementary Table S2, Fig. 3). All studies integrated clinical score and PRS through either a logistic or a Cox model. The results were similar to those obtained for breast cancer. For clinical scores comprising many relevant non-genetic risk factors, the PRS added only little in terms of performance, as was exemplified by type 2 diabetes. For diseases with only few known risk factors, such as Parkinson disease, the PRS alone was found to be as efficient as the combined score.

The combined score for type 2 diabetes (Lall et al. 2017) yielded the best diagnostic performance of all diseases and diagnostic models. Here, the AUC of the clinical score, including body mass index (BMI), sex and age, was increased from 0.775 to 0.8 by the respective PRS. When BMI was removed from the clinical score, the impact of the PRS was even more pronounced, changing the AUC from 0.699 to 0.74. A similar improvement was observed by Black et al. (2020) for a prostate cancer score comprising age and family history. There, the AUC increased from 0.6 to 0.64, but the utility of the combined score was questionable because the PRS alone already yielded an AUC of 0.64.

### Clinical application of PRSs

One of the obvious clinical applications of PRSs would be the estimation of the residual lifetime risk for diseases for which risk-adjusted screening is meaningful. A prime example in this regard is breast cancer, where the American Cancer Society recommends screening by magnetic resonance imaging (MRI) when the lifetime risk exceeds 20–25% (Saslow et al. 2007). Using a PRS comprising 24 SNPs, Li et al. (2017) monitored 2599 healthy women for an average of 7.4 years and calculated the residual lifetime risk from both BOADICEA and BOADICEA + PRS. Adopting a risk threshold of 20% (or 25%, henceforth referred to in brackets), some 35.7% (42.2%) of women placed above the threshold by BODICEA alone fell below it with BOADICEA + PRS whereas 15.7% (10.7%) of women placed above the threshold by BOADICEA + PRS fell below it with BOADICEA alone. In total, combination of BOADICEA with the PRS changed the screening recommendation for 23% (14%) of participants.

Similarly, Lakeman et al. (2019) calculated a PRS comprising 161 SNPs for 323 cases and 262 controls from 101 high-risk breast cancer families lacking *BRCA* mutations. The authors then investigated how their risk classifications according to the NCCN (National Comprehensive Cancer Network, USA), NICE (National Institute for Health and Care Excellence, UK) and the IKNL (Netherlands Comprehensive Cancer Organisation, NL) guidelines changed by combining BOADICEA with the PRS. Whilst the NCCN distinguishes between low and high risk by a threshold of 20%, NICE and IKNL distinguish between low, moderate and high risk with thresholds of 17% and 30%, and 20% and 30%, respectively. Combining BOADICEA with the PRS changed the screening recommendation for 11.5% of women for NCCN, 14.7% for NICE, and 19.8% for IKNL. Cases were more frequently shifted to a higher risk category than controls.

For breast cancer, consideration of a PRS has already made its way into clinical practice. Thus, the Centre for Familial Breast and Ovarian Cancer in Cologne, Germany, offers CanRisk (Carver et al. 2021), a CE-certified web tool to calculate BOADICEA version 6 which also includes the PRS of Mavaddat et al. comprising 313 SNPs (Lee et al. 2019; Mavaddat et al. 2019). To study the extent to which women would actually want to know their genetic risk for breast cancer and how they would handle this knowledge, Yanes et al. (2021) asked 208 healthy Australian women for their decision at baseline and followed them for 12 months. If a participant had agreed, genetic health professionals informed her about her individual 62 SNP PRS, which was then classified as either low, moderate or high, followed by a comparison to the overall breast cancer risk in the population. Some 165 women (79%) agreed to be informed, and 91% of them still remembered their PRS category after 12 months. A majority of those who were told their PRS (57%) had no regrets about their decision. Participants with a high PRS regretted their decision slightly more often than those with a low PRS, but not as much as those who refused to know their PRS altogether. However, knowledge of the PRS had no influence upon the willingness to be screened.

In a study of prostate cancer, Huynh-Le et al. (2020) defined risk-equivalent age groups based upon a PRS comprising 54 SNPs (Seibert et al. 2018). Notably, using the prior disease risk at 50 years of age as a reference, it turned out that men with a PRS in the 1^st^ percentile do not reach this risk before the age of 60 whereas those with a PRS in the 99^th^ percentile already do so at 41. Risk-equivalent age also had a strong influence upon the positive predictive value (PPV) of the prostate-specific antigen screening test. Between 55 and 64 years of age, the PPV is 21% for those with a risk-equivalent age in their actual age range, 12% for those with a risk-equivalent age below 55 years, and 40% when the risk-equivalent age is > 65 years.

Another potential application of PRSs is the stratification of drug treatment. In a clinical study by Damask et al. (2020), 11,953 patients previously hospitalized for myocardial infarction or unstable angina were treated with either alirocumab, a PCSK9 inhibitor, or placebo. Participants were followed-up for MACE (major adverse cardiovascular event) for a median of 2.8 years. In addition, they were genotyped to allow computation of a coronary artery disease-specific PRS. The incidence of MACE, which comprises death of coronary heart disease, nonfatal myocardial infarction, ischemic stroke, or unstable angina requiring hospitalization (Bosco et al. 2021), was 17.4% in the top PRS decile of the placebo group, compared to 11.5% in the lowest decile. At 11.4% (top) and 10.0% (lowest), by contrast, the two incidence values were found to differ much less in the verum group. The authors concluded that patients with a high genetic risk might benefit more from alirocumab treatment than those with a low genetic risk. A study by Marston et al. (2020) yielded similar results for evolocumab, another PCSK9 inhibitor used to treat cardiovascular disease. In this study, some 14,298 patients with atherosclerotic cardiovascular disease were treated with either verum or placebo and were followed for a median of 2.3 years. Among patients with a low PRS-based genetic risk, and without any clinical risk factors, the hazard ratio (HR) (verum versus placebo) for a major vascular event was virtually unity. Among patients with multiple clinical risk factors, however, the HR equaled 0.87 and the number needed to treat (NNT) 71 when the genetic risk was low, whereas HR = 0.69 and NNT = 25 when the genetic risk was high.

Prevention is one of the three key areas of the 2020 “Genome UK” program, which deals with PRSs as well (HM Government 2020). Researchers can access the available scores, and participants are provided an opportunity of personal risk assessment. Over a period of 10 years, Genome UK is set to help evolving the National Health Service from disease detection and treatment to disease prediction and prevention. A public health and screening system is envisaged to this end that uses genomics to intensify screening and intervention for individuals with high disease risk, including the development and transition into practical use of risk prediction tools comprising both genetic and non-genomic factors. Not least, the project is hoped to generate evidence whether PRSs can be used in large-scale health services and whether they help to reduce the burden on healthcare. Similar goals are also pursued by another UK program, called “Our Future Health”, that plans to recruit up to five million representative adults for translational research on new tools and strategies for diagnostics, prevention and treatment. Notably, one of the primary objectives of the program is to validate PRS-based predictive models of health and disease (Our Future Health 2022).

The above developments notwithstanding, randomized studies systematically comparing PRS-informed screening or treatment with standard-of-care are still necessary to be able to judge the true benefit of including PRSs into clinical practice. While some such studies have started recently (Hao et al. 2022), no results are available as yet.

## Discussion

### Selection of studies

One important finding of our literature-based survey of PRSs was the frequent lack of comparability between the measures of PRS performance used in different studies. Such variety is not surprising in view of the many ways in which the capability of a diagnostic or prognostic marker can be measured. In addition to the AUC and C statistic considered in our study, other useful metrics include the coefficient of determination, the odds ratio, the relative risk and the hazard ratio. What is more, the performance measures were often not calculated for a PRS alone, but in combination with other covariates such as age, gender or the genetic background. Since such a combination with other information can strongly increase the diagnostic or prognostic capability ascribed to a PRS, the results are not comparable to those obtained without covariates. In fact, many studies had to be excluded from our survey for this reason. We may thus conclude that the development and implementation of reporting guidelines for PRS studies would greatly improve their practical benefit (Wand et al. 2021).

A limitation of our survey has been that it was confined to PRSs developed predominantly in samples of European ancestry. Our results therefore cannot be transferred immediately to other ethnicities (Duncan et al. 2019). Moreover, the study by Gola et al. (2020) served to highlight that even applying one and the same PRS to different European cohorts may yield considerably different results. Since most GWAS underlying PRSs have been performed in European ancestry populations, there is thus a need for more diverse data to ensure equitable participation in the research and health progress potentially arising from the use of PRSs (Caliebe et al. 2022).

### Performance evaluation

For the PRSs covered by our study, AUC was generally between 0.65 and 0.70 in the corresponding validation studies. A notable exception is type 1 diabetes for which AUC was around 0.90. This result may point towards the specific genetic architecture of this disease. Indeed, according to Noble and Valdes (2011), 40–50% of the heritability of type 1 diabetes is explained by the HLA locus alone. In line with this, a PRS based upon five HLA region SNPs only already achieved an AUC of 0.87 (Oram et al. 2016; Sharp et al. 2019). The addition of 25 non-HLA SNPs increased the value to 0.89. Using 35 HLA and 32 non-HLA SNPs, the AUC value was found to be as high as 0.92, with 0.90 achieved by the HLA SNPs alone, and 0.75 by the non-HLA-SNPs alone (Sharp et al. 2019).

We assessed the performance of a PRS by its AUC. However, while the AUC is a popular measure in diagnostic studies, other criteria have been proposed particularly for PRSs. For example, individuals in the extreme upper quantiles of a PRS distribution are often thought to benefit most from the clinical use of the PRS because their odds ratios come close to those of monogenic diseases (Khera et al. 2018). Early identification of such individuals would allow timely prevention, if available. Notwithstanding the appeal of the approach, it must be noted that putting a focus upon extreme values of a PRS would greatly limit its overall clinical utility because only a small fraction of the population would get something out of having the PRS measured.

### Clinical applications

Since the positive predictive value of a PRS is typically low in diagnostic settings (Ala-Korpela & Holmes 2020; Koch et al. 2021; Wald & Old 2019), the same is inevitably true in the prognostic context, i.e. many false-positive results are to be expected when a PRS is applied to predict future disease. Therefore, it would usually remain unclear which meaningful consequences can be drawn from a positive prognostic result (i.e. a high PRS). The incentive for a lifestyle change or other preventive treatment would likely be low if only a few percent of individuals with a positive test result indeed developed the disease of interest later in life. Moreover, it is by no means clear for individuals with a high PRS whether the effect of such a change would be as expected. On the contrary, a strong role of genetics in the etiology of a disease may even imply that those with a high PRSs benefit less from a lifestyle change than those with a low PRS. In addition, the benefit of earlier, or more frequent, screening has to be weighed against potential side effects such as, for example, radiation damage, over-screening, over-diagnosis and psychological distress.

Use of a PRS for prognostic testing as a means to improve screening efficiency currently seems most promising for breast cancer and prostate cancer. However, while PRS-based changes in screening recommendations may be expected to occur frequently for these entities, it has not been investigated yet whether such changes have any meaningful clinical consequences, including the avoidance of unnecessary screening, the earlier detection of disease or, ultimately, a reduction in cancer-related deaths. As regards diseases other than cancer, we noted that PRSs for type 1 diabetes have exceptionally high AUC and are thus potentially useful in cases where clinical signs of risk are ambiguous (Padilla-Martinez et al. 2020). For type 2 diabetes, in contrast, PRSs promise no relevant improvement over the use of known clinical risk factors alone, similar to other diseases.

Another obstacle to the translation of PRSs into clinical routine is the difficult interpretation of an actual PRS value. The value by itself has no straightforward meaning. Even standardized PRS values, or population quantiles, are only meaningful when gauged against cases and, at best, lead only to relative risks, i.e. a high PRS value is not tantamount to a high absolute disease risk and vice versa. To convey this discrepancy to medical practitioners and patients is a huge challenge, adding another argument to the need for randomized clinical studies to compare PRS-informed decisions with standard-of-care. Some such trials have already started (Hao et al. 2022) with the goal to avoid or delay disease occurrence, or to improve treatment without generating unwanted side effects.

Finally, the introduction of PRSs into clinical practice will also have health-economic implications. The incremental costs caused by deriving PRSs must be justified by the added value which, in turn, will depend upon the disease and practical application in question. Unfortunately, important parameters such as the number needed to treat or to screen, are usually not provided by PRS publications. One notable exception is a study by Marston et al. (2020), who reported the numbers needed to treat with evolocumab for cardiovascular disease patients, stratified by their PRS value. Before using PRSs like this in clinical practice, however, the numbers would have to be related to the costs of treatment and screening. Since the latter likely vary between countries and therapeutic settings, further research is required to measure the true value of adding a PRS to existing screening or treatment protocols.

### PRSs in research

While their application in clinical routine may still be contentious, PRSs clearly bear great potential in medical research. For example, applying a disease-specific PRS to another condition may shed light upon common genetic etiologies. Early on, Purcell et al. (2009) developed a PRS for schizophrenia and applied it to patients with bipolar disorder, revealing that the PRS for the former disease explained some of the risk for the latter. The same approach has also been useful to define and study disease subtypes. For example, Stahl et al. (2019) showed that a PRS for schizophrenia was significantly increased in patients with type 1 bipolar disorder, compared to type 2 bipolar disorder. A PRS for depression, on the other hand, was higher in those with type 2 bipolar disorder than in type 1 patients.

Other PRS-related research was focused upon the expressivity and the symptoms of diseases. In a study by Ruderfer et al. (2018), a schizophrenia PRS turned out to be increased in bipolar patients when psychotic features were present, but not when they were lacking, while a PRS for bipolar disorder was higher in schizophrenia patients with manic symptoms than in those without. The authors expressed the hope that, by identifying the polygenetic components of the different symptoms of schizophrenia and bipolar disorder, more conclusions can be drawn about possible treatments. By applying a PRS for Alzheimer disease to Parkinson disease patients with and without hallucinations, Kusters et al. (2020) showed that the latter symptom is associated with the same genetic factors, especially APOE variation, that are responsible for the cognitive deficits in Alzheimer patients.

PRSs may also help to gain new insights into the variability in age-at-onset of monogenic diseases. In a study by Fahed et al. (2020), PRSs for coronary heart disease, breast cancer and colorectal carcinoma were applied to their monogenic counterparts, namely familial hypercholesterolemia, hereditary breast and ovarian cancer, and Lynch syndrome. For all three diseases, the risk up to the age of 75 of carriers of monogenic mutations was positively correlated with the respective PRS. The authors concluded that the polygenic background leading to the common complex form of a disease may influence one or more of the molecular pathways affected by corresponding monogenetic mutations.

Applying a PRS to another disease can also augment studies of the effectiveness of drug treatment. Several PRSs, including those for attention deficit hyperactivity disorder and coronary artery disease, were found to be associated with the treatment success of antidepressants in major depressive disorder (Amare et al. 2019; Fabbri et al. 2021; Meerman et al. 2022). Higher PRS values consistently co-occurred with more treatment-resistant symptoms. Finally, PRSs can be tools to study the interaction between genetics and environment or lifestyle. This was exemplified by a study by Ye et al. (2021) that revealed a statistical interaction on triglyceride levels between lifestyle on the one hand, and a PRS for coronary artery diseases, atrial fibrillation and type 2 diabetes on the other. Bolli et al. (2021) discovered an interaction on coronary artery disease risk between a disease-specific PRS and low-density lipoprotein cholesterol level.

### Outlook

It is likely that, in the future, PRSs will be proposed more and more often for the prognosis and diagnosis of common complex diseases, and for treatment decision making. This growing popularity of PRSs is not only due to the fact that the required SNP genotyping has become increasingly cheaper. Obviously, PRSs represent a rather efficient, and hence attractive, way to take the genetic background of patients into account in efforts to improve the performance of the predictive statistical models used in medical care.

### Conclusions

- Current PRSs have limited capability for individual risk prediction.
- Exceptions are likely due to a specific genetic architecture of the disease in question, such as the strong effect of the HLA locus in the case of type 1 diabetes.
- Adding a PRS to a clinical score increases the AUC by about 10% on average, depending upon disease, clinical score and age.
- Some PRSs may be clinically useful for screening and therapeutic decision making.
- Assessment of the cost-effectiveness of a PRS in clinical practice requires estimation, in randomized trials, of the corresponding number needed to screen, or to treat.

### Supplementary Information

Below is the link to the electronic supplementary material.

Supplementary file1 (DOCX 120 KB)

## Data Availability

The authors confirm that the data supporting the findings of this study are available within the article [and/or] its supplementary materials.
