# Supplementary material for: Clinical utility of polygenic risk scores: a critical 2023 appraisal
Source: J Community Genet. 2023 May 3;14(5):471–87. doi: 10.1007/s12687-023-00645-z (PMC10576695; doi:10.1007/s12687-023-00645-z)
Supplement: Supplementary file 1 — Supplementary file1 (DOCX 120 KB) [file 12687_2023_645_MOESM1_ESM.docx]

**Clinical Utility of Polygenic Risk Scores: A Critical 2022 Appraisal
Supplementary Information**

Sebastian Koch^1^, Jörg Schmidtke^2,3^, Michael Krawczak^1^, Amke Caliebe^1*^

*1 Institut für Medizinische Informatik und Statistik, Christian-Albrechts-Universität zu Kiel, Universitätsklinikum Schleswig-Holstein Campus Kiel, Kiel, Germany*

*2 Amedes MVZ Wagnerstibbe, Hannover, Germany*

*3 Institut für Humangenetik, Medizinische Hochschule Hannover, Hannover, Germany*

* corresponding author; caliebe@medinfo.uni-kiel.de

**Table S1** Exemplary combinations of clinical breast cancer scores and risk factors with PRSs

| **Score** | **PRS only** | **Score only** | **PRS + Score** | 1. **test setting** 2. **measure** 3. **combined model** | **Reference** |
| --- | --- | --- | --- | --- | --- |
| BOADICEA  (<50 years) | 0.63^2^ | 0.691^3^ | 0.704 | 1. diagnostic 2. AUC 3. integration in BOADICEA formula | Choudhury et al. (2021)  PRS: Mavaddat et al. (2019) |
| BOADICEA + age + risk factors^1^  (<50 years) |  |  | 0.697 |  |  |
| BOADICEA  (>50 years) |  | 0.568^3^ | 0.624 |  |  |
| BOADICEA + age + risk factors^1^  (>50 years) |  |  | 0.646 |  |  |
| BCRAT  (<50 years, no BRCA1/2) | 0.61 | 0.64 | 0.66 | 1. diagnostic 2. AUC^4^ 3. multiplicative | Dite et al. (2016)  PRS:  Mavaddat et al. (2015) |
| BOADICEA  (<50 years, no BRCA1/2) |  | 0.66 | 0.7 |  |  |
| BRCAPRO  (<50 years, no BRCA1/2) |  | 0.65 | 0.69 |  |  |
| IBIS  (<50 years, no BRCA1/2) |  | 0.57 | 0.632 |  |  |
| Several risk factors^5^ | 0.583 | 0.564 | 0.604 | 1. diagnostic 2. AUC^6^ 3. logistic model | Husing et al. (2012) |
| BOADICEA + age  (< 70 years) | 0.632  (< 60 years)  0.673  (60-70 years) | 0.531 | 0.636 | 1. prognostic 2. C statistic   c) integration in BOADICEA formula | Lakeman et al. (2020)  PRS: Mavaddat (2019) |
| BOADICEA + age + risk factors^7^  (< 70 years) |  | 0.558 | 0.653 |  |  |
| BCRAT^8^ | 0.627 | 0.677 | 0.708 | 1. prognostic 2. C statistic 3. Cox model | Lall et al. (2019)  PRS: Mavaddatet al. (2015) |
| BCSC | 0.68 | 0.66 | 0.69 | 1. diagnostic 2. AUC 3. multiplicative | Vachon et al. (2015) |
| IBIS + MD | NA | 0.64 | 0.67 | 1. prognostic 2. AUC^9^ 3. multiplicative | Van Veen et al. (2018) |
| BCRAT + MD  (pre-menopausal) | NA | 0.559 | 0.641 | 1. diagnostic 2. AUC^4^ 3. logistic model | Zhang et al. (2018) |
| BCRAT + MD + hormones^10^  (post-menopausal, without HT) |  | 0.555 | 0.66 |  |  |
| BCRAT + MD + Prolactin  (post-menopausal, with HT) |  | 0.58 | 0.649 |  |  |

PRS: polygenic risk score, BCRAT: Breast Cancer Risk Assessment Tool, BOADICEA: Breast and Ovarian Analysis of Disease Incidence and Carrier Estimation Algorithm, IBIS: International Breast Cancer Intervention Study, MD: mammographic density, HT: hormone therapy, BCSC: Breast Cancer Surveillance Consortium risk-prediction model, AUC: area under the receiver operating characteristic curve, NA: not available. ^1^age at menarche, at first live birth and at menopause, number of live births, use of oral contraception and of HT, body mass index (BMI), height, alcohol consumption, history of benign breast disease and breast cancer family history in first degree relatives, ^2^from Mavaddat et al. (2019), ^3^for BOADICEA only, ^4^age-adjusted, ^5^age at menarche, at first birth and at menopause, and count of full term pregnancies, BMI, alcohol consumption, smoking and HT, ^6^age and cohort adjusted, ^7^age at menarche, at first birth and at menopause, number of children, use of oral contraception and of HT, BMI, height, alcohol consumption, ^8^with predictors age, ethnicity, age at menarche and at full term pregnancy, ^9^outcome incidence of Parkinson disease within follow-up time of UK Biobank, ^10^testosterone, estrone sulfate and prolactin

**Table S2** Exemplary combinations of risk factors and clinical scores with PRSs for selected diseases, other than breast cancer

| **Disease** | **Score** | **PRS only** | **Score only** | **PRS + Score** | 1. **test setting** 2. **measure** 3. **combined model** | **Reference** |
| --- | --- | --- | --- | --- | --- | --- |
| Coronary artery disease | Framingham Risk Score  (age < 75) | - | 0.731 | 0.742 | 1. prognostic 2. C statistic^1^ 3. Cox model | Abraham et al. (2016) |
|  | ACC/AHA13  (age < 75) | - | 0.729 | 0.739 |  |  |
|  | age + sex | 0.61 | 0.73 | 0.76 | 1. prognostic 2. C statistic 3. Cox model | Elliott et al. (2020) |
|  | QRISK3 |  | 0.78 | 0.79 |  |  |
| Parkinson disease | PREDICT-PD | 0.577 | 0.759 | 0.767 | 1. prognostic 2. AUC^2^ 3. logistic model | Jacobs et al. (2020) |
| Prostate cancer | age + family history | 0.64 | 0.60^3^ | 0.64 | 1. diagnostic 2. AUC 3. logistic model | Black et al. (2020) |
| Type 2 diabetes | sex +age  (age 40-79, prevalent T2D) | - | 0.699 | 0.74 | 1. diagnostic (prevalent T2D), prognostic (incident T2D) 2. AUC (prevalent), C statistic (incident) 3. logistic regression (prevalent)   Cox regression (incident) | Lall et al. (2017) |
|  | BMI + sex + age  (age 40-79, prevalent T2D) |  | 0.775 | 0.8 |  |  |
|  | BMI + sex +age + other risk factors^4^  (age 35-79, incident T2D) |  | 0.777 | 0.79 |  |  |

PRS: polygenic risk score, BMI: body mass index, AUC: area under the receiver operating characteristic curve, ACC/AHA13: American College of Cardiologists/American Heart Association 2013 risk score, T2D: type 2 diabetes. ^1^cohort-adjusted and sex-stratified, ^2^incidence of Parkinson disease within follow-up time of UK Biobank, adjusted for genetic principal components, ^3^adjusted for family history, ^4^risk factors: waist circumference, waist-hip ratio, history of hypertension, history of high blood glucose, physical activity level, smoking, fruit and vegetable

**References**

Abraham G, Havulinna AS, Bhalala OG, Byars SG, De Livera AM, Yetukuri L et al (2016) Genomic prediction of coronary heart disease. Eur Heart J 37:3267-3278. doi:10.1093/eurheartj/ehw450

Black MH, Li S, LaDuca H, Lo MT, Chen J, Hoiness R et al (2020) Validation of a prostate cancer polygenic risk score. Prostate 80:1314-1321. doi:10.1002/pros.24058

Choudhury PP, Brook MN, Hurson AN, Lee A, Mulder CV, Coulson P et al (2021) Comparative validation of the BOADICEA and Tyrer-Cuzick breast cancer risk models incorporating classical risk factors and polygenic risk in a population-based prospective cohort of women of European ancestry. Breast Cancer Res 23. doi:10.1186/s13058-021-01399-7

Dite GS, MacInnis RJ, Bickerstaffe A, Dowty JG, Allman R, Apicella C et al (2016) Breast cancer risk prediction using clinical models and 77 independent risk-associated SNPs for women aged under 50 years: Australian Breast Cancer Family Registry. Cancer Epidemiol Biomarkers Prev 25:359-365. doi:10.1158/1055-9965.EPI-15-0838

Elliott J, Bodinier B, Bond TA, Chadeau-Hyam M, Evangelou E, Moons KGM et al (2020) Predictive accuracy of a polygenic risk score-enhanced prediction model vs a clinical risk score for coronary artery disease. Jama-J Am Med Assoc 323:636-645. doi:10.1001/jama.2019.22241

Husing A, Canzian F, Beckmann L, Garcia-Closas M, Diver WR, Thun MJ et al (2012) Prediction of breast cancer risk by genetic risk factors, overall and by hormone receptor status. J Med Genet 49:601-608. doi:10.1136/jmedgenet-2011-100716

Jacobs BM, Belete D, Bestwick J, Blauwendraat C, Bandres-Ciga S, Heilbron K et al (2020) Parkinson's disease determinants, prediction and gene-environment interactions in the UK Biobank. J Neurol Neurosur Ps 91:1046-1054. doi:10.1136/jnnp-2020-323646

Lakeman IMM, Rodriguez-Girondo M, Lee A, Ruiter R, Stricker BH, Wijnant SRA et al (2020) Validation of the BOADICEA model and a 313-variant polygenic risk score for breast cancer risk prediction in a Dutch prospective cohort. Genet Med 22:1803-1811. doi:10.1038/s41436-020-0884-4

Lall K, Lepamets M, Palover M, Esko T, Metspalu A, Tonisson N, Padrik P, Magi R,Fischer K (2019) Polygenic prediction of breast cancer: comparison of genetic predictors and implications for risk stratification. BMC Cancer 19. doi:10.1186/s12885-019-5783-1

Lall K, Magi R, Morris A, Metspalu A,Fischer K (2017) Personalized risk prediction for type 2 diabetes: the potential of genetic risk scores. Genet Med 19:322-329. doi:10.1038/gim.2016.103

Mavaddat N, Michailidou K, Dennis J, Lush M, Fachal L, Lee A et al (2019) Polygenic risk scores for prediction of breast cancer and breast cancer subtypes. Am J Hum Genet 104:21-34. doi:10.1016/j.ajhg.2018.11.002

Mavaddat N, Pharoah PD, Michailidou K, Tyrer J, Brook MN, Bolla MK et al (2015) Prediction of breast cancer risk based on profiling with common genetic variants. J Natl Cancer Inst 107. doi:10.1093/jnci/djv036

Vachon CM, Pankratz VS, Scott CG, Haeberle L, Ziv E, Jensen MR et al (2015) The Contributions of Breast Density and Common Genetic Variation to Breast Cancer Risk. Jnci-J Natl Cancer I 107. doi:10.1093/jnci/dju397

van Veen EM, Brentnall AR, Byers H, Harkness EF, Astley SM, Sampson S et al (2018) Use of Single-Nucleotide Polymorphisms and Mammographic Density Plus Classic Risk Factors for Breast Cancer Risk Prediction. Jama Oncol 4:476-482. doi:10.1001/jamaoncol.2017.4881

Zhang X, Rice M, Tworoger SS, Rosner BA, Eliassen AH, Tamimi RM et al (2018) Addition of a polygenic risk score, mammographic density, and endogenous hormones to existing breast cancer risk prediction models: A nested case-control study. PLoS Med 15:e1002644. doi:10.1371/journal.pmed.1002644
